# Muscle synergy patterns as altered coordination strategies in individuals with chronic low back pain: a cross-sectional study

**DOI:** 10.1186/s12984-023-01190-z

**Published:** 2023-05-31

**Authors:** Hiroki Saito, Hikaru Yokoyama, Atsushi Sasaki, Kimitaka Nakazawa

**Affiliations:** 1 Graduate School of Arts and Sciences, Department of Life Sciences, The University of Tokyo, Tokyo, Japan; 2 Department of Physical Therapy, Tokyo University of Technology, Tokyo, Japan; 3 Institute of Engineering, Tokyo University of Agriculture and Technology, Tokyo, Japan; 4 Graduate School of Engineering Science, Department of Mechanical Science and Bioengineering, Osaka University, Osaka, Japan; 5 Japan Society for the Promotion of Science, Tokyo, Japan

**Keywords:** Trunk muscle synergies, Low back pain, Variability, Motor control

## Abstract

**Background:**

Chronic low back pain (CLBP) is a highly prevalent disease with poorly understood underlying mechanisms. In particular, altered trunk muscle coordination in response to specific trunk tasks remains largely unknown.

**Methods:**

We investigated the muscle synergies during 11 trunk movement and stability tasks in 15 healthy individuals (8 females and 7 males, aged 21. 3 (20.1–22.8) ± 0.6 years) and in 15 CLBP participants (8 females and 7 males, aged 20. 9 (20.2–22.6) ± 0.7 years) by recording the surface electromyographic activities of 12 back and abdominal muscles (six muscles unilaterally). Non-negative matrix factorization was performed to extract the muscle synergies.

**Results:**

We found six trunk muscle synergies and temporal patterns in both groups. The high similarity of the trunk synergies and temporal patterns in the groups suggests that both groups share the common feature of the trunk coordination strategy. We also found that trunk synergies related to the lumbar erector spinae showed lower variability in the CLBP group. This may reflect the impaired back muscles that reshape the trunk synergies in the fixed structure of CLBP. Furthermore, the higher variability of trunk synergies in the other muscle regions such as in the latissimus dorsi and oblique externus, which were activated in trunk stability tasks in the CLBP group, represented more individual motor strategies when the trunk tasks were highly demanding.

**Conclusion:**

Our work provides the first demonstration that individual modular organization is fine-tuned while preserving the overall structures of trunk synergies and temporal patterns in the presence of persistent CLBP.

**Supplementary Information:**

The online version contains supplementary material available at 10.1186/s12984-023-01190-z.

## Introduction

Low back pain (LBP) is the most common cause of disability worldwide [1]. It decreases the patients quality of life and contributes to enormous direct health care costs and lost productivity costs [2–4]. In fact, experience of persistent/chronic LBP (CLBP) is largely associated with these costs [4]. The determination of the underlying mechanism of CLBP through the use of neurophysiological indices such as electromyographic (EMG) recordings has been the subject of intense investigations aiming to identify altered trunk motor control strategies [5, 6]. This endeavor is critical for providing efficient biomarkers as indicators of pathological processes and responses to therapeutic intervention [7].

Previous studies have shown changes in trunk muscle activation in individuals with CLBP [5, 8, 9]. However, the responses to CLBP are highly variable and often contradictory [5]. One of the reasons for this is related to the issue that EMG can only record from a few of the trunk muscles, owing to the limited information that can be obtained from the EMG recordings. Fundamentally, many spinal and abdominal muscles act as functional units for trunk stability and movement [10]. Theses trunk muscles present bilateral and unilateral connectivity defined by the anatomical constraints (e.g., common origin and insertion of muscles) and the neural circuitry that projects to functional groups of muscles for dynamic motor control [11]. Therefore, assessing multiple patterns of trunk EMG activity in an integrative fashion is required to accurately identify the overall picture of altered motor control in CLBP [12].

The application of dimensionality reduction algorithms, such as non-negative matrix factorization (NMF) [13], to EMG activities has been largely utilized to investigate coordination patterns or muscle synergies formed by multiple muscles activated in synchrony during many human and animal behaviors [14, 15]. This is based on the premise that the central nervous system (CNS) relies on a limited number of muscle synergies to simplify movement production [16, 17]. NMF employs linear extraction and separates EMG matrices into time-invariant weighted activations of a group of muscles (muscle weighting components) and time-variant activation profiles (temporal pattern components) [16, 18, 19]. For this method, large-scale and high-dimensional EMG data recorded during a variety of motor tasks are essential, as muscle synergies are the fundamental building blocks of neural constraints [20]; thus, it may address the question of how the CNS adapts in the presence of CLBP [12]. Furthermore, incorporating many trunk motor tasks is beneficial, as this increases the possibility of revealing the motor control strategies in CLBP that may have altered trunk motor control during different motor tasks [21]. It also helps to interpret the impact of this altered control during many daily living and sports activities [22]. We have previously revealed several trunk muscles synergies with unilateral and bilateral patterns of muscle activations, underlying locomotion and stability motor behaviors in healthy individuals [22]. We further found that theses functional trunk muscle synergies can be extracted from the EMG data of 11 trunk movement and stability tasks, which aimed to effectively capture the altered trunk muscle synergies in a clinical scenario such as CLBP [12]. In this context, the trunk motor tasks were symmetrically designed, and the EMG data of each task was time-interpolated to have the same data points, which would minimize the possibility that the extracted synergies were biased toward any certain directions.

The altered trunk muscle synergies underlying a variety of functional trunk motor tasks that involve multiple trunk muscles in those with CLBP remain undefined. Thus, in the present study, we examined trunk muscle synergies and temporal patterns in healthy participants and in individuals with CLBP during 11 trunk motor tasks [12]. We aimed to reveal altered trunk neuromuscular control in CLBP participants by comparing muscle synergy structures between healthy participants and patients with CLBP [23, 24]. Second, we investigated the differences in the variability of trunk muscle synergies and temporal patterns between the groups. Assessments of variability reflect the degree of motor abundance, which indicates the likelihood of achieving motor outputs with different recruitment muscle patterns [25]. Thus, these help assess the stereotypes or diverse features of trunk motor control strategies in CLBP participants compared to non-CLBP participants.

## Methods

### Participants

Fifteen individuals with CLBP participated in this study (8 females and 7 males, mean age 20. 9 [20.2–22.6] ± standard deviation [SD] 0.7 years). Individuals with CLBP were considered to have experienced non-specific LBP for longer than 3 months, to have experienced continuous LBP for the last 3 months, or to have had periods of symptom aggravation and remission in the last 6 months [26]. Fifteen age- and sex-matched healthy individuals were also recruited as the control group (8 females and 7 males, aged 21. 3 [20.1–22.8] ± 0.6 years). Pain-free individuals (non-CLBP) participated if they had no relevant history of LBP that limited their motor function and/or required treatment from a health professional.

From both the non-CLBP and CLBP groups, we excluded participants who had any of the following: (1) circulatory, neurological, or respiratory diseases; (2) recent or current pregnancies; (3) previous spinal surgery; (4) back pain with radiating pain to their legs; and (5) those who had undergone current treatment for LBP by health care professionals. We also excluded individuals with CLBP if they had experienced an acute “flare up” of their LBP as a result of performing motor tasks, as well as if they had been taking any medications such as opioids, anticonvulsives, antidepressants, or regularly high-dose nonsteroidal anti-inflammatory drugs (NSAIDs), as evidence has indicated that taking medications may modulate trunk motor control [27]. The study was conducted in accordance with the Declaration of Helsinki and its later amendments and was approved by the local ethics committee of the University of Tokyo.

### Questionnaires

A questionnaire was administered to obtain information on participant demographics, duration, average intensity, and area of pain. Participants with CLBP completed the Roland-Morris Questionnaire (RMQ) to measure LBP-related disability [28]. The Tampa Scale for Kinesiophobia (TSK) was used to assess fear avoidance and fear avoidance beliefs [29, 30]. Participants with CLBP also completed the short form of the Orebro Musculoskeletal Pain Questionnaire (OMPQ) to assess psychological factors that specifically focused on predicting long-term pain, disability, and sick leave [31, 32], as well as the Pain Self-Efficacy Questionnaire (PSEQ) to measure the patients’ self-efficacy for pain [33].

### Experimental procedures

**Fig. 1:**
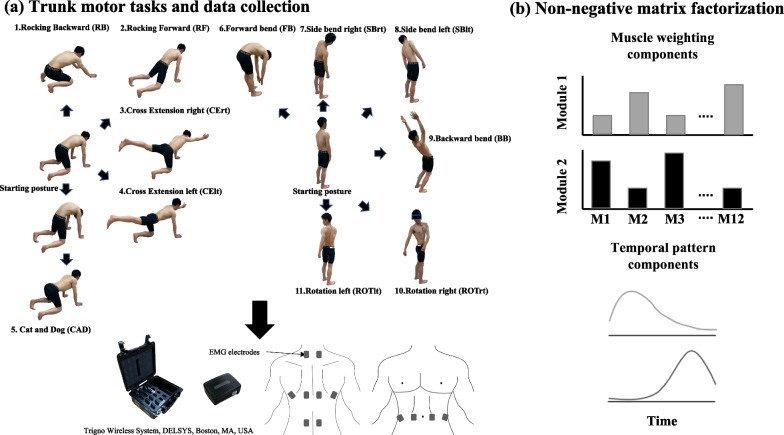
A schematic of the experimental design. **a** The 11 trunk movements and stability tasks. EMG data (12 trunk muscles) were recorded during these tasks. Each task was repeated 8 times. **b** Non-negative matrix factorization was applied to the concatenated EMG of all 11 tasks to decompose it into the muscle weighting components (e.g., 12 muscles and two modules) according to the temporal pattern components. EMG, electromyography

Participants were asked to freely perform the 11 trunk-related movement and stability tasks described in Fig. 1a. Each motor task has largely been utilized in research and clinical fields to evaluate and improve motor control in individuals with LBP [21, 34–36]. Detailed descriptions of theses motor tasks can be found elsewhere [12]. The starting point of a task was initiated with the verbal cue “go” when the examiner manually pressed the electrical trigger once [22, 37]. After the participants completed the tasks and returned to a starting posture for approximately 1 s, the examiner manually pressed the electrical trigger twice with the verbal cue “end” to define the end of the movement [22, 37]. Each task was repeated eight times, and the order of the tasks was randomly assigned.

### Data collection

Bilateral surface EMG data were recorded from six spinal and abdominal muscle groups: rectus abdominis (RA) (3 cm lateral to umbilicus) [38], oblique externus (OE) (15 cm lateral to umbilicus) [39], erector spinae at L3 (ESL3) (3 cm lateral to the L3 spinous process) [38], erector spinae at Th9 (EST9) (5 cm lateral to the T9 spinous process) [40], erector spinae at Th1 (EST1) (5 cm lateral to the T1 spinous process) [40], and latissimus dorsi (LD) (lateral to T9 over the muscle belly) [39]. Surface EMG activity was recorded using a wireless EMG system (Trigno Wireless System, DELSYS, Boston, MA, USA). Each electrode had an inter-electrode spacing of 10 mm. The EMG signals were bandpass filtered (20–450 Hz), amplified (with a 300-gain preamplifier), and sampled at 1000 Hz using an analog-to-digital converter (Power lab/16SP, AD Instruments, Australia).

### EMG processing

Raw EMG signals were high-pass filtered at 30 Hz using a fourth-order Butterworth filter to remove motion artifacts [41]. The signals were then demeaned, full-wave-rectified and low-pass filtered at 10 Hz using a fourth-order Butterworth filter [42]. The smoothed EMG envelopes were time-interpolated using linear interpolation to generate 200 time points between the start and end points for each trial so that the EMG data of each trial equally contributed to the extracted muscle synergies.

To facilitate the extraction of muscle synergies representing neural constraints on movement, the incorporation of large-scale and high-dimensional EMG data, including a variety of tasks with loosely constrained scenarios, is suggested [22, 23, 37, 43]. Thus, we created concatenated EMG matrices from all 11 trunk motor tasks to obtain an “all-task” EMG matrix for each subject (that is, the matrix was composed of 12 muscles × the no. of repetitions (8) × 200 samples from the 11 single-task EMG matrices) to extract the trunk muscle synergies underlying the 11 trunk tasks. The EMG recording from each muscle was normalized to the maximum amplitude across all tasks. Then, each muscle vector in the data matrix was standardized to have unit variance, such that the activity in each muscle was equally weighted.

Because of the nature of LBP, each LBP participant may have differences in the dominant area of LBP. To avoid biases due to the differences in the dominant sides, which may affect the between group comparison of muscle synergies, the more affected area was set to the same sides in all LBP participants. Specifically, in EMG data for individuals with CLBP, more and less affected sides were treated as the left and right sides, respectively. Accordingly, the order of tasks that had asymmetric patterns between sides also needed to be adjusted. The pairs of cross extension right (task 3) and left tasks (task 4), side bend right (task 7) and left tasks (task 8), rotation right (task 10) and left tasks (task 11) were the same motor tasks with asymmetric movements, which lead to asymmetric muscle activation patterns between right and left sides. Therefore, the EMG data that accounted for these pairs in the LBP participants who had adjusted the sides were interchanged when creating the all-task EMG matrix.

### Muscle synergy extraction

NMF based on the multiplicative update rules was applied to the all-task EMG matrix to extract the muscle synergies (Fig. 1b). NMF has previously been described as a linear decomposition technique [13, 18] according to Eq. (1):

1 $$M=W\cdot C+e$$

where *M* (*m* × *t* matrix, where *m* is the number of muscles and *t* is the number of samples, i.e., spatiotemporal profiles of muscle activity) is a linear combination of muscle weighting components *W* (*m* × *n* matrix, where n is the number of muscle synergies), temporal pattern components *C* (*n* × *t* matrix), and *e* is the residual error matrix. The initialization of W and C was set randomly [13]. We applied NMF to extract each possible *n* value from 1 to 12 from each dataset. To estimate the optimal number of muscle synergies, the variance accounted for (VAF) by the reconstructed EMG (*M*) was calculated at each iteration [44]. The VAF was defined as 100× the square of the uncentered Pearson’s correlation coefficient [44, 45]. Considering the local minima inherent in NMF, each synergy extraction was repeated 50 times, and the VAF was calculated for each possible number of synergies. The iterations with the highest VAF were maintained [22, 37, 46, 47]. VAFs > 90% were used to identify the optimal number of synergies commonly used in the literature [22, 37, 48–51]. It was suggested that the criterion VAF > 90% ensures a sufficient representation of the data [42], although this is still debated [52, 53]. To facilitate the comparisons between groups, we used the same number of muscle synergies as the rounded mean number of synergies across participants for further analysis [46, 54].

### Sorting synergies based on similarity indices

Following the application of NMF, the order of the muscle weighting components and temporal pattern components may have been inter-versed between participants. Thus, functional sorting was necessary. First, we grouped the extracted muscle synergies using a hierarchical cluster analysis implemented by MATLAB (“linkage” function, MathWorks, Inc., Natick, MA, USA) [55]. The cluster analysis partitioned the hierarchical cluster tree with the minimum number of clusters for which there was no more than one synergy from the same participant in each cluster [56]. The muscle weighting components and their corresponding temporal pattern components within the cluster were then averaged (synergy cluster centroids). Next, we sorted the relatively subject-invariant cluster centroids in muscle weighting components (defined as having synergies from ≥ 1/3 of the participants) in the CLBP group based on the centroids in the non-CLBP group [57, 58]. Specifically, the scalar product was computed for every possible pair of synergy cluster centroids in muscle weighting components between groups. Then, we selected the pair with the highest value of similarity, and the synergy cluster centroids involved in that pair were removed from the set. The highest similarity value among the remaining sets was chosen, and the pair was removed until all synergy cluster centroids were appropriately matched. This resulted in similar muscle weighting components, and the corresponding temporal pattern components were of the same order in both groups. We also computed the *r*_max_ coefficient using the cross-correlation function for the cluster centroids of the temporal pattern components between the groups [51, 55].

### Variability within clusters

For each group, we computed the similarity between participants (intra-cluster similarity) by averaging the similarity values of all pairwise dot products between the muscle weighting components and the r_max_ coefficient between temporal pattern components in each cluster as variability within clusters.

### Statistical analysis

We compared the number of muscle synergies and the VAF between the non-CLBP and CLBP groups. For the between-group comparisons of synergy structures, the extracted muscle synergies need to be matched [46, 59]. Thus, the comparison was performed if the similarities of each between-group pair of muscle weighting components defined by the scalar product, and temporal pattern components defined by the r_max_ coefficient were > 0.8 [57]. Specifically, we compared individual weightings of each muscle synergy vector to investigate the differences in the contribution of muscles. For each temporal pattern component, we first divided temporal patterns into 1600 time points of each task (i.e., 200 timepoints × 8 repetitions for each task) as temporal pattern components composed of the data of 11 trunk motor tasks. We then calculated the average of the maximum values of 8 repetitions in each bin. The values at each time point represented the maximum activation levels in each task for each temporal pattern component. Then, the values between groups were compared to investigate the effects of CLBP on activation profiles for each task. Lastly, we compared each intra-cluster similarity of the muscle weighting components, and the temporal pattern components between groups. The values were compared using the Mann–Whitney *U* test for between group comparison because a normal distribution was not observed in the data (tested using the Shapiro–Wilk test).

We also investigated the relationship between the synergy similarity to the control group (non-CLBP) and several clinical scores in CLBP to assess the effects of clinical impairments on muscle synergy structure. Specifically, we calculated the Spearman’s rank correlation between the average similarity of the centroid pairs of muscle synergy (all Ws and all Cs, respectively) components in non-CLBP and muscle weighting components in each individual with CLBP, as well as the clinical scores including duration of pain (month), pain intensity (NRS), TSK, RMQ, OREBRO and PSEQ. Spearman’s rank correlation was used because a normal distribution was not observed in the data (tested using the Shapiro–Wilk test).

The significance level for all tests was set at *p* = 0.05. The *p* values obtained from all tests were corrected using the Bonferroni correction for multiple comparisons [60]. When there was significant difference between the groups, effect sizes were calculated using Cohen’s d [61].

## Results

### Participants

The demographic and clinical characteristics of the non-CLBP and CLBP groups are shown in Table 1. Individuals with CLBP rated their current pain intensity as 3.1 ± 1.0, while the non-CLBP group reported no pain at the time of the experiment. In terms of the participants’ dominant pain areas, 10 of the 15 CLBP individuals had left-side dominant LBP and 5 CLBP individuals had right-side dominant LBP. Thus, the EMG data on the right side in these five LBP participants were replaced with the EMG data on left side vice versa, so that all LBP participants had the affected area on the left side of trunk muscles (see in the method section).

**Table 1:** The demographic and clinical characteristics of the non-Chron Low Back Pain (CLBP) and CLBP groups

Characteristics	Non-CLBP group (n = 15)	CLBP group (n = 15)
Age (years)	21.3 (± 0.6)	20.9 (± 0.7)
Height	166.5 (± 10.6)	164.8 (± 9.1)
Weight	58.6 (± 10.8)	58.8 (± 9.0)
Sex (female/male)	8/7	8/7
Area of dominant pain (right/left)	–	5/10
Duration of pain (months)	–	45.7 (± 23.5)
Average pain intensity (NRS)	–	3.2 (± 1.0)
RMQ	–	3.6 (± 2.6)
TSK	–	39.8 (± 9.5)
OREBRO	–	30.4 (± 12.7)
PSEQ	–	32.8 (± 7.5)

*NRS* The numerical rating scale; *RMQ* The Roland-Morris Questionnaire; *TSK* The Tampa Scale for Kinesiophobia; *OREBRO* The Örebro Musculoskeletal Pain Questionnaire; *PSEQ* The Pain Self-Efficacy Questionnaire

### EMG patterns of 11 trunk motor task in the non-CLBP and CLBP groups

**Fig. 2:**
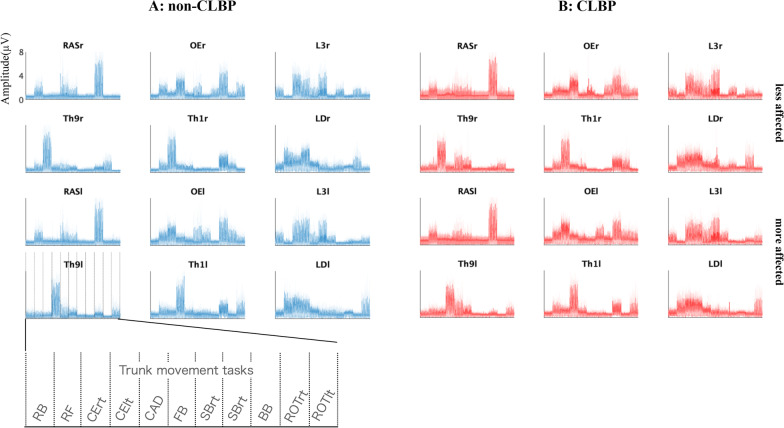
EMG patterns of 11 trunk motor task in the non-CLBP and CLBP group. The concatenated EMG envelops of 11 trunk motor tasks in all participants with and without CLBP. The mean of the EMG envelopes for all Non-LBP and LBP participants are plotted as a line and the standard deviation as a shading around it. The amplitude is normalized by the maximum value for each trunk muscle over all 11 tasks. Then, each muscle vector in the data matrix was standardized to have unit variance, such that the activity in each muscle was equally weighted. Of note, the five LBP participants had the more affected area of LBP on the right side and the remaining ten were on the left side. Thus, the EMG data on the right side in these five LBP participants were replaced with the EMG data on left side vice versa, so that all LBP participants had the affected area on the left side of trunk muscles (please see the “Methods” section)

Figure 2 shows the concatenated EMG envelops of 11 trunk motor tasks in all participants with and without CLBP. The mean of the EMG envelopes for all non-CLBP and CLBP participants are plotted as a line and the standard deviation as a shading around it.

Based on visual inspection, symmetrical activation patterns between right and left sides were identified in the RAS, OE and LD. In contrast, asymmetrical activation patterns were found in the Th9 and Th1. Visual inspection also shows no major difference in activation patterns between groups.

### Trunk muscle synergies in the non-CLBP and CLBP groups

**Fig. 3:**
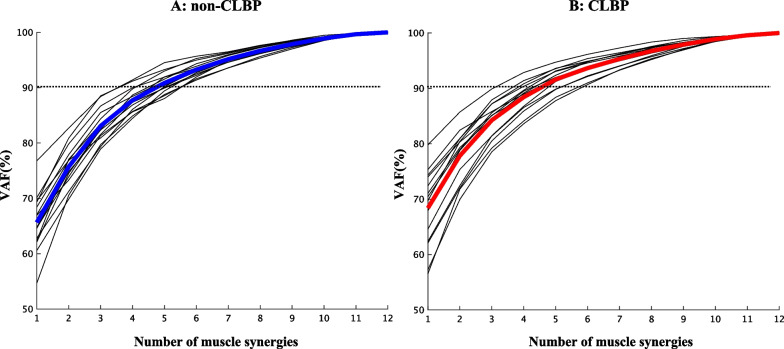
Individual (thin line) and mean participant (think lines) percentages of the variability accounted for (VAF). The left and right panels indicate the VAFs of the non-CLBP and CLBP groups, respectively. The horizontal dashed lines indicate the thresholds used to determine the number of extracted trunk muscle synergies

Figure 3 shows the VAF for each muscle synergy in the non-CLBP and CLBP groups. The extracted number of synergies were 5.20 ± 0.77 (mean ± SD) and 5.00 ± 0.75 in the non-CLBP and CLBP groups, respectively. There was no significant difference in the number of participants between the groups (*p* = 0.916). For further between-group comparison of muscle synergy structures, we extracted five muscle synergies as the rounded mean in both groups. When five synergies were extracted, the VAFs were 90.85% ± 1.86 and 91.62% ± 1.96 in the non-CLBP and CLBP groups, respectively. There was no significant difference in the VAFs between the groups (*p* = 0.260).

**Fig. 4:**
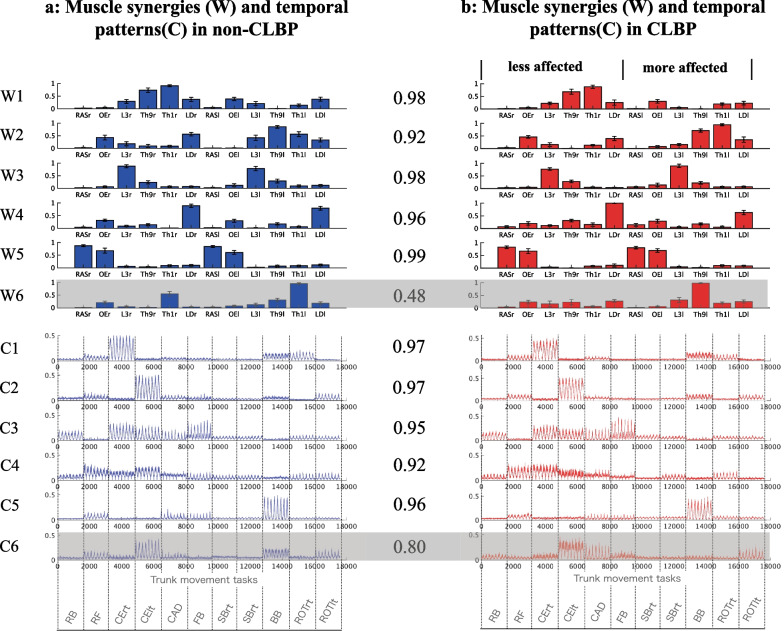
The trunk muscle synergies in the non-CLBP and CLBP groups. **a** Averages of the six synergy cluster centroids (mean ± SE) and the corresponding temporal patterns (the mean is plotted as a line and the SE is shaded around the line) of the non-CLBP group (n = 15). **b** Averages of the six synergy cluster centroids of the CLBP group (n = 15). Each of the six synergies in the non-CLBP group was matched to a synergy from the CLBP group to acquire the highest scalar product (the values are shown between the pairs). *RB* Rocking Backward, *RF* Rocking Forward, *CErt* Cross Extension Right, *Celt* Cross Extension Left, *CAD* Cat and Dog, *FB* Forward bend, *SBrt* Side bend right, *SBlt* Side bend left, *BB* Backward bend, *ROTrt* Rotation right, *ROTlt* Rotation left

Figure 4 shows the cluster centroids of muscle weighting components and the cluster centroids of the temporal pattern components in individuals with and without CLBP. Five centroids of the trunk synergies (W1 to W5 and C1 to C5) had similarity values > 0.9, and W6 and C6 had similarity values of 0.48 and 0.80, respectively.

W1 mainly represents the activation of the right Th1, Th9, and LD muscles along with the activation of the left side OE. C1 shows that W1 is largely activated during the cross-extension of the right side. W2 represents the asymmetric pattern of W1. It represents the activation of the left Th1, Th9, and LD muscles with the activation of the right side OE and mainly activates the cross-extension of the left side.

W3 mainly involves the bilateral activation of the L3 muscles. W3 works during the rocking backward, cross extension of both sides, and forward bend tasks. Similarly, W4 mainly represents the bilateral LD muscles and OE. W4 is activated during the forward rocking and the cross extension of both sides tasks. W5 mainly involves bilateral activation of the RAS and OE muscles. W5 is engaged during the backward-bend task. Lastly, W6 in the non-CLBP group involves bilateral activations of Th1 during the cross-extension of the left side, and backward bend task. In contrast, W6 in the CLBP group involved left side activation of Th9 during the cross-extension of the left side.

### The between-group comparison: muscle synergy vectors, temporal patterns and intra-cluster similarity

We performed between-group comparisons in individual weightings of muscle synergy vectors, temporal pattern components, and intra-cluster similarity for W1 to W5, and C1 to C5. Of note, W6 and C6 were removed from the comparison based on the criteria described in the methods section. The results showed there were no significant differences in individual weightings across muscle synergy vectors (W1 to W5) and in each bin of activation levels of temporal patterns (C1 to C5) between groups (*p* > 0.05).

**Fig. 5:**
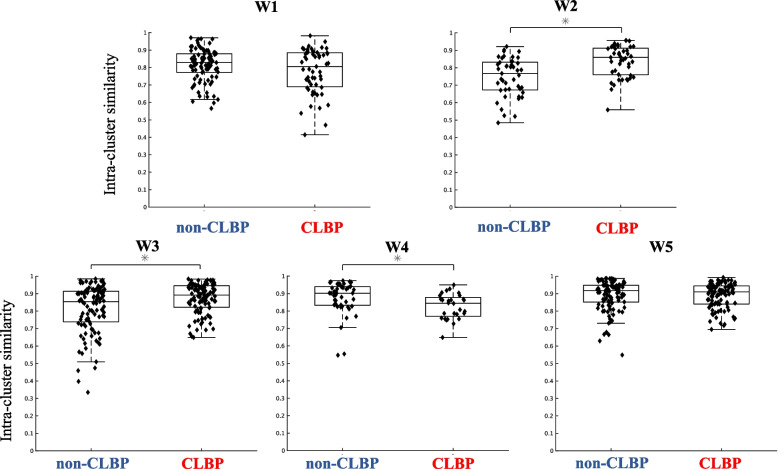
Intra-cluster similarity in the trunk muscle synergies (W1 to W5) between the non-CLBP and CLBP groups. The intra-cluster similarity of the muscle weighting components in W2 and W3 were significantly higher in the CLBP group than in the non-CLBP group (W2: *p* = 0.00006, d = 0.89; W3: *p* = 0.0012, d = 0.53). The similarity in W4 was significantly lower in the CLBP group than in the non-CLBP group (*p* = 0.0021, d = 0.57). There was no significant difference in W1 and W5 between the groups (W1: *p* = 0.241, W5: *p* = 0.599)

**Fig. 6:**
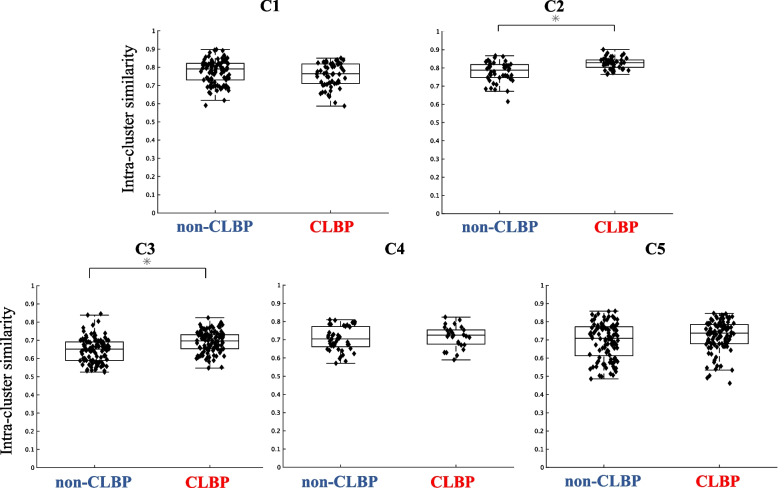
Intra-cluster similarity values of the trunk muscle synergies (C1 to C5) between the non-CLBP and CLBP groups. The intra-cluster similarity of the temporal pattern components in C2, C3 and C5 were significantly higher in the CLBP group than in the non-CLBP group (C2: *p* = 0.000009, d = 1.07; C3: *p* = 0.0000006, d = 0.70; C5: *p* = 0.047, d = 0.30). There was no significant difference in C1 and C4 between the groups (C1: *p* = 0.152; C4: *p* = 0.385)

Figures 5 and 6 represent a comparison of the intra-cluster similarity of the trunk muscle synergies and temporal patterns between the non-CLBP and CLBP groups. As shown in Fig. 5, the intra-cluster similarity of the muscle weighting components in W2 and W3 were significantly higher in the CLBP group than in the non-CLBP group (W2: *p* = 0.00006, *d* = 0.89; W3: *p* = 0.0012, *d* = 0.53). The similarity in W4 was significantly lower in the CLBP group than in the non-CLBP group (*p* = 0.0021, *d* = 0.57). There was no significant difference in W1 and W5 between the groups (W1: *p* = 0.241, W5: *p* = 0.599). As shown in Fig. 6, the intra-cluster similarity of the temporal pattern components in C2, C3 and C5 were significantly higher in the CLBP group than in the non-CLBP group (C2: *p* = 0.000009, *d* = 1.07; C3: *p* = 0.0000006, *d* = 0.70; C5: *p* = 0.047, d = 0.30). There was no significant difference in C1 and C4 between the groups (C1: *p* = 0.152; C4: *p* = 0.385).

There was no significant correlation between the averages of synergy similarity (W and C) to the centroids in the non-CLBP group and several clinical scores in the CLBP group (*p* > 0.05). The results are presented in the Supplementary information.

## Discussion

To the best of our knowledge, this is the first study to investigate trunk muscle synergies during a variety of trunk motor tasks in CLBP participants. On average, the trunk muscle weighting components and temporal pattern components were similar between the non-CLBP and CLBP groups except for W6. Furthermore, there were no significant differences in individual weightings across matched muscle weighting components (W1 to W5) and the activation levels in each task of matched temporal pattern components (C1 to C5) between groups. These findings suggest that overall, both groups share the common feature of the trunk coordination strategy. Furthermore, individuals with CLBP were characterized by higher and lower inter-participant variability in their trunk synergies compared to those without CLBP. This finding indicates that trunk muscle synergies and their temporal patterns were fine-tuned and reflected impaired trunk muscle structures and activations, the latter of which is considered a potential mechanism for the development and persistence of CLBP.

In our analysis, we incorporated 11 trunk motor tasks that can effectively reveal the motor control deficits in individuals with LBP [21, 34–36]. However, our results showed that the number of synergies and the VAFs of the five synergies extracted did not significantly differ between the groups. Overall, the gross muscle synergies and their temporal patterns were strikingly similar between the groups. Thus, the overall structure of the muscle synergies and their temporal patterns were maintained in the presence of CLBP. This is in line with previous studies of upper limb synergies in patients with stroke during a variety of upper limb movements, which found that both the unaffected and affected upper limbs were strikingly similar to each other, despite there being differences in motor performance between the limbs and different degrees of motor cerebral lesions [24]. Because previous and current studies utilized the EMG data with higher variability in many motor tasks, the extracted synergies may represent neural constraints on movement and provide robust features of the synergies, despite the presence of underlying disease [20]. In addition, the current study also found there were no significant differences in individual weightings across muscle synergy vectors (W1 to W5) and each bin of activation levels of temporal patterns (C1 to C5) between groups. In contrast, previous studies found that the acute or sub-acute pain conditions such as experimentally induced pain using hypertonic saline [62, 63] and at postoperative pain at 1 month following lumber surgery [64], lead to the significant changes in activation profiles in painful muscles [62–65] and the reduced number of synergies [63, 64]. Potentially, this inconsistency is related to the presence of LBP conditions. This suggests that acute and high-level painful conditions lead to the exploration of new adaptive motor patterns to avoid pain provocation [66], while the chronicity with low level disability and pain in the current study minimize the effect of LBP on the motor control strategy. Lastly, the non-CLBP group showed W6 was activated with bilateral patterns of Th1 in the cross-extension left task, which may contribute to stabilize the upper trunk during these tasks. However, the LBP group produced different muscle weighting components with left side activations of Th9 (W6) during the cross-extension left task as shown in C6. Of note, in EMG data of all individuals with CLBP, more and less affected sides were treated as the left and right sides, respectively (please see the “Methods” section). Because the left cross-extension task may increase the mechanical demand on the left side of the trunk (Fig. 1a), it may be possible that when the mechanical demand to the left side of the back regions is relatively high, LBP participants whose left side is more affected tend to produce additional back muscle activations to complete a task. However, this excessive activation of trunk muscles would increase the spinal load and may contribute to the persistence of LBP [67]. This is in the line with a previous study investigating the muscle synergies during a lifting task in the CLBP group [68]. The study showed increased activations of synergies with back muscle coactivation patterns, which is considered as protective behaviors in individuals with CLBP [68].

We also found significant differences in the inter-participant variability of both the trunk muscle synergies and temporal patterns in the CLBP group compared to those in the non-CLBP group. This provides evidence that the individual modular organizations were at least fine-tuned for motor adaptation to LBP while preserving the overall structures of the trunk synergies and temporal patterns. Specifically, our results showed that the unilateral (W2) and bilateral (W3) erector spinae muscle patterns presented higher intra-cluster similarity in the CLBP group than in the non-CLBP group. Altered trunk muscles have been proposed to underpin LBP development and recurrence [6]. In particular, when LBP persists, the ongoing effects of pain and inflammatory mechanisms lead to altered back and abdominal muscle structures, including atrophy, muscle fiber changes, and fatty inflation [69]. Thus, the common features of the alterations may lead to the reshaping of muscle weighting components at painful region (W2 and W3) in similar structures among the participants in the CLBP group.

Similarly, the intra-cluster similarity values of the corresponding C2, C3 and C5 in the CLBP group were also significantly higher than those in the non-CLBP group. In healthy individuals, it is evident that temporal patterns present more variable features due to individualized motor development to fit the anthropometry and muscle architecture of the individual [70]. These features represent motor abundance, which is the possibility of achieving motor outputs with different recruitment muscle patterns [25]. Interestingly, the higher variability of temporal patterns was even more apparent in athletes with many years of training and a high level of expertise in maximizing their motor performance [70]. In contrast, the decreased variability (higher intra-cluster similarity) in the temporal patterns of the CLBP group may represent the loss of motor abundance. As demonstrated in Table 1, the CLBP group in the current study showed a relatively high fear of movement as assessed by the TSK (> 37 points) [30]. Previous studies showed that individuals with CLBP tend to move slower and have decreased spinal movement variability associated with LBP and the fear of movement [5, 21, 71]. Moreover, Fig. 4 shows that the activation levels in C2 and C3 were higher during the spinal stability tasks (task 3, 4 and 5) and the forward bend task (task 6), which are considered as provocative motor tasks in individual with LBP [72]. Thus, it is likely that LBP with the fear of movement provoked by the higher demanding tasks lead to the decreased variability in the LBP group, reflecting similar temporal profiles between CLBP participants.

Our results also found a significantly lower intra-cluster similarity in the W4 in the CLBP group compared to in the non-CLBP group. W4 is composed of the bilateral LD and OE, which may function to control and stabilize the upper trunk by sharing rib insertions [73]. Figure 4 shows that W4 are activated mainly during trunk stability tasks, such as rocking forward task and the cross extension task, which require the trunk to maintain its posture and prepare to counteract the reactive spine [69]. Thus, we speculated that the challenging biomechanical demands facilitated the individualized changes in the muscle weighting components in the CLBP group compared to in the non-CLBP group, resulting in higher variability of W4 in individuals with CLBP. The CNS employing a more individualized motor strategy can be considered as an adaptive strategy to counteract the loss of optimal variability at the painful site (W2 and W3), as it minimizes the pain intensity and level of disability in the current CLBP population, despite the longer duration of LBP (Table 1). However, whether the observed strategy in the CLBP group represents an adaptive or maladaptive strategy could not be determined owing to the cross-sectional design of the current study.

There are several limitations in the interpretation of our results. First, the recordings of muscle activity were performed only on the superficial trunk muscles that could be accessed via the surface EMG devices used in the current study. However, a number of studies have suggested that the deeper layers of the trunk muscles, such as the multifidus and transversus abdominis, are also associated with persistence of LBP [74–76]. Second, we did not measure any kinematic data, which limited the interpretability of the possible interaction of the task complexity, clinical impairments, and motor outputs such as muscle synergies, trunk angles and velocity. Furthermore, a relatively young CLBP group with low disability and pain intensity participated in the current study, which may result in the changes in the extracted synergies in presence of CLBP being minimized, and no significant correlation was observed between the synergy similarity to the centroids in the non-CLBP group and several clinical scores of the LBP group (*p* > 0.05) (Additional file 1). Such issues could result in their LBP being inadequately represented, rendering it inappropriate to generalize the results to the entire CLBP population. Overall, it can be speculated that in future research, recording both the superficial and deep trunk muscles using surface and intramuscular electrodes in a more greatly disabled CLBP group would enable us to more clearly comprehend the underlying mechanisms of persistent of LBP based on the biopsychological frameworks [1].

## Conclusion

This study found that the individual modular organization is fine-tuned while preserving the overall structures of trunk synergies and temporal patterns in the presence of persistent CLBP. Altered inter-participant variability of trunk muscle synergies in CLBP reflected the impaired trunk muscles and the adaptations to the specific mechanical demands.

## Supplementary Information

**Additional file 1: Figure S1.** Correlations between the averages similarity between the centroids of muscle weighting components and each muscle weighting components in CLBP group, and clinical scores. Above: Spearman’s rank correlation coefficientand its significancebetween similarity and clinical scales. Black dots show similarity valuesand clinical scoresfor CLBP participantsand the blue lines represent the regression lines. **Figure S2. **Correlations between the averages similarity between the centroids of temporal pattern components and each temporal pattern components in CLBP group, and clinical scores. Above: Spearman’s rank correlation coefficientand its significancebetween similarity and clinical scales. Black dots show similarity valuesand clinical scoresfor CLBP participantsand the blue lines represent the regression lines.

## Data Availability

The datasets used and/or analyzed during the current study are available from the corresponding author upon reasonable request.
