# Correction to: Muscle synergy patterns as altered coordination strategies in individuals with chronic low back pain: a cross-sectional study

**DOI:** 10.1186/s12984-023-01210-y

**Published:** 2023-07-14

**Authors:** Hiroki Saito, Hikaru Yokoyama, Atsushi Sasaki, Kimitaka Nakazawa

**Affiliations:** 1 Graduate School of Arts and Sciences, Department of Life Sciences, The University of Tokyo, Tokyo, Japan; 2 Department of Physical Therapy, Tokyo University of Technology, Tokyo, Japan; 3 Institute of Engineering, Tokyo University of Agriculture and Technology, Tokyo, Japan; 4 Graduate School of Engineering Science, Department of Mechanical Science and Bioengineering, Osaka University, Osaka, Japan; 5 Japan Society for the Promotion of Science, Tokyo, Japan

Following publication of the original article [1], Fig. 6 in the original version of this article has been replaced and the figure has shown below:

**Fig. 6:**
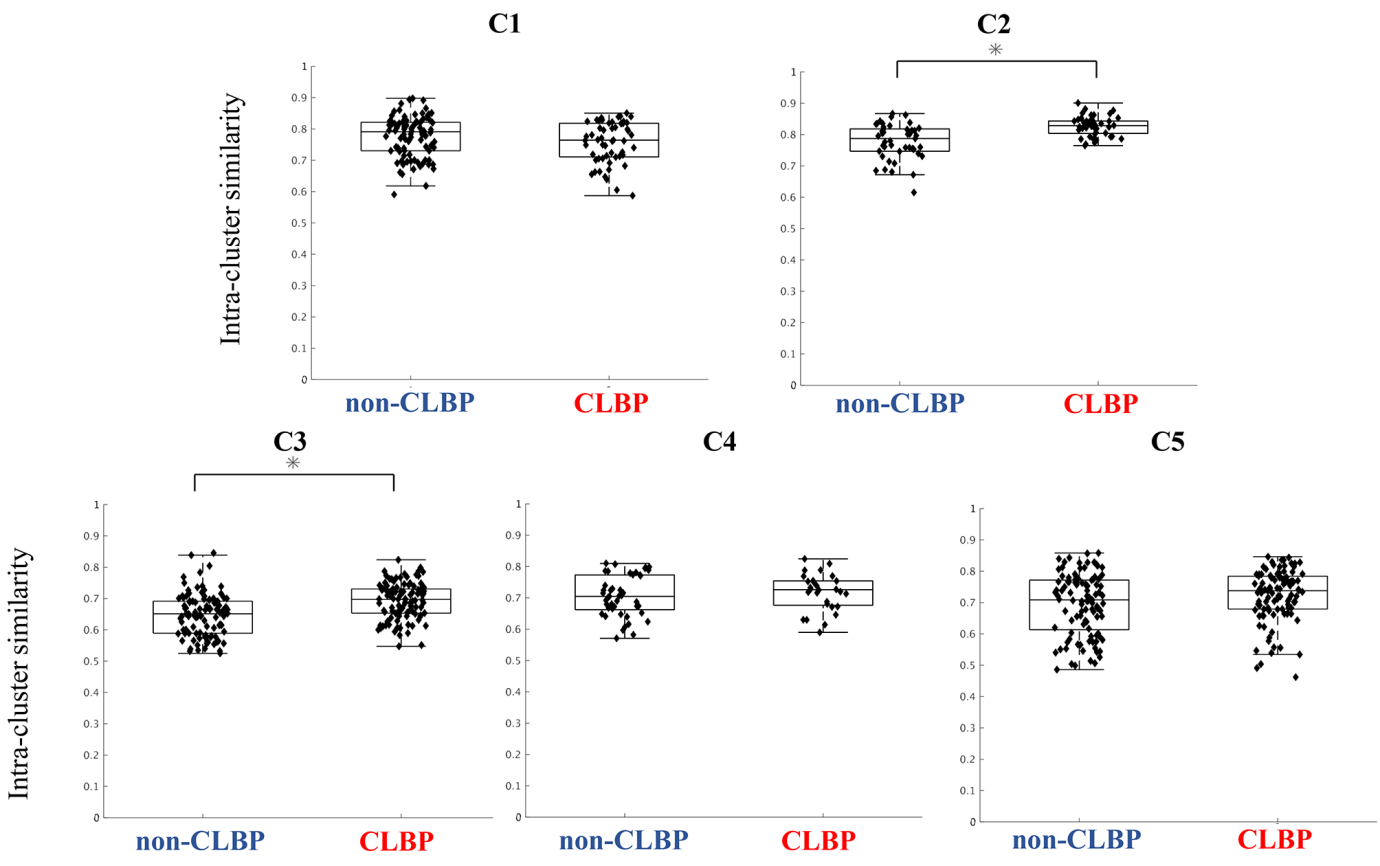
Intra-cluster similarity values of the trunk muscle synergies (C1 to C5) between the non-CLBP and CLBP groups. The intra-cluster similarity of the temporal pattern components in C2, C3 and C5 were significantly higher in the CLBP group than in the non-CLBP group (C2: *p* = 0.000009, d = 1.07; C3: *p* = 0.0000006, d = 0.70; C5: *p* = 0.047, d = 0.30). There was no significant difference in C1 and C4 between the groups (C1: *p* = 0.152; C4: *p* = 0.385)

The original article has been corrected.

## References

[1] Saito H, Yokoyama H, Sasaki A et al. Muscle synergy patterns as altered coordination strategies in individuals with chronic low back pain: a cross-sectional study. J NeuroEngineering Rehabil. 2023;20:69. 10.1186/s12984-023-01190-z PMC10230697 37259142

