# Supplementary material for: Muscle synergy patterns as altered coordination strategies in individuals with chronic low back pain: a cross-sectional study
Source: J Neuroeng Rehabil. 2023 May 31;20:69. doi: 10.1186/s12984-023-01190-z (PMC10230697; doi:10.1186/s12984-023-01190-z)
Supplement: Supplementary file 1 — Additional file 1: Figure S1. Correlations between the averages similarity between the centroids of muscle weighting components and each muscle weighting components in CLBP group, and clinical scores. Above: Spearman’s rank correlation coefficientand its significancebetween similarity and clinical scales. Black dots show similarity valuesand clinical scoresfor CLBP participantsand the blue lines represent the regression lines. Figure S2. Correlations between the averages similarity between the centroids of temporal pattern components and each temporal pattern components in CLBP group, and clinical scores. Above: Spearman’s rank correlation coefficientand its significancebetween similarity and clinical scales. Black dots show similarity valuesand clinical scoresfor CLBP participantsand the blue lines represent the regression lines. [file 12984_2023_1190_MOESM1_ESM.docx]

**Additional Information**

**Title:** Muscle synergy patterns as altered coordination strategies in individuals with chronic low back pain: a cross-sectional study

Hiroki Saito^1,2^, Hikaru Yokoyama^3*^, Atsushi Sasaki^4, 5^, Kimitaka Nakazawa^1^

^1^ Graduate School of Arts and Sciences, Department of Life Sciences, The University of Tokyo, Tokyo, Japan

^2^ Department of Physical Therapy, Tokyo University of Technology, Tokyo, Japan

^3^ Institute of Engineering, Tokyo University of Agriculture and Technology, Tokyo, Japan

^4^ Graduate School of Engineering Science, Department of Mechanical Science and Bioengineering, Osaka University,

^5^ Japan Society for the Promotion of Science, Tokyo, Japan

Correspondence to: Hikaru Yokoyama

Institute of Engineering, Tokyo University of Agriculture and Technology, Tokyo, Japan

Email: [h-yokoyama@go.tuat.ac.jp](mailto:h-yokoyama@go.tuat.ac.jp)

**Figure S1.** Correlations between the averages similarity between the centroids of muscle weighting components and each muscle weighting components in CLBP group, and clinical scores. Above: Spearman’s rank correlation coefficient (*r*) and its significance (*p*-value) between similarity and clinical scales. Black dots show similarity values (X axis) and clinical scores (Y axis) for CLBP participants (n = 15) and the blue lines represent the regression lines.

**Figure S2.** Correlations between the averages similarity between the centroids of temporal pattern components and each temporal pattern components in CLBP group, and clinical scores. Above: Spearman’s rank correlation coefficient (*r*) and its significance (*p*-value) between similarity and clinical scales. Black dots show similarity values (X axis) and clinical scores (Y axis) for CLBP participants (n = 15) and the blue lines represent the regression lines.
